# Elemental Changes in Riesling Wines Following Controlled Apple Juice Addition: An ICP–OES-Based Model Study

**DOI:** 10.3390/molecules31203655

**Published:** 2026-10-13

**Authors:** Magdalena Gajek, Joanna Ścibiorek, Małgorzata Iwona Szynkowska-Jóźwik

**Affiliations:** Institute of General and Ecological Chemistry, Faculty of Chemistry, Lodz University of Technology, Zeromskiego 116, 90-924 Lodz, Poland; joanna.scibiorek2@gmail.com (J.Ś.); malgorzata.szynkowska@p.lodz.pl (M.I.S.-J.)

**Keywords:** wine adulteration, Riesling wine, apple juice, ICP–OES, multi-element analysis, chemometric analysis, principal component analysis, nonparametric statistics

## Abstract

Wine adulteration with inexpensive fruit juices remains a significant authenticity issue. Apple juice, due to its neutral sensory properties, can be added to white wines without visual detection. While elemental analysis is widely applied in wine authentication, its suitability for detecting adulteration with low-mineral juices has not been systematically evaluated. In this study, 60 commercial white wines representing seven grape varieties and 45 Riesling samples intentionally adulterated with 5%, 10%, and 20% (*v*/*v*) apple juice were analyzed for 24 elements using ICP–OES. The data were evaluated using nonparametric statistical tests, including Mann–Whitney U, Wilcoxon signed-rank, and Friedman tests, with Holm correction for multiple comparisons, as well as Pearson correlation analysis and principal component analysis. Apple juice addition caused a decrease in most evaluated elements, especially Ca, Mg, Mn, P, S, and Sr, reflecting dilution of the wine matrix, whereas Na showed an opposite trend, particularly at the highest adulteration level. The reduced PCA model based on selected diagnostic variables provided clearer visualization of the adulteration-related pattern than the model based on all variables and highlighted the separation of samples adulterated at 20% (*v*/*v*). Apple juice addition also increased wine pH. The results demonstrate that ICP–OES data combined with statistical and chemometric analysis can reveal compositional changes associated with apple juice adulteration in a Riesling wine model.

## 1. Introduction

Wine is one of the most popular alcoholic beverages worldwide, and its significance on the Polish market has been steadily increasing. According to data published by Polish. Wine, the average annual consumption of wine and mead in Poland reached 6.8 L per person in 2023, confirming a continuous upward trend compared with previous years [1]. Between 2018 and 2022, the total value of wine sales in Poland increased by approximately 28% [2]. Wine is also becoming more common in on-premise consumption, with around 42% of Polish consumers choosing it when drinking outside their homes [3]. Although the Polish wine market remains dominated by red wines, there is a clear and growing interest in white wines, which are gradually gaining a larger share of sales. Consumer preferences are shifting toward dry styles and well-known international grape varieties such as Chardonnay and Sauvignon Blanc are among the most frequently chosen [4]. Riesling, which is gaining in popularity, is a variety traditionally cultivated in cooler regions of Europe, including Poland. Wines produced from Riesling are characterized by their high acidity, fresh aroma, and considerable aging potential, making them an increasingly appreciated choice among both producers and consumers [5].

Wine is a chemically complex beverage composed of water, ethanol, and residual sugars, as well as a broad range of organic and inorganic constituents, including amino acids, organic acids, polyphenolic compounds, vitamins, and mineral elements [6,7]. It cannot be overlooked that wine consumption has been associated with certain health benefits, partly due to the presence of bioactive compounds and essential trace elements that may contribute to the human dietary intake [6,8]. However, wine may also contain undesirable concentrations of toxic metals and metalloids such as arsenic (As), cadmium (Cd), and lead (Pb), which can pose potential health risks [6]. Moreover, the mineral composition of wine can significantly affect its sensory characteristics—aroma, color, and mouthfeel—as well as important physicochemical parameters such as alcohol content and total acidity.

The elemental profile of each wine is influenced by a wide variety of factors. These are associated with every stage of the winemaking process—from grape cultivation, through production and maturation, to transportation and storage. They include the grape variety, soil type, and climatic conditions characteristic of the cultivation region, the use of plant protection agents, as well as technological aspects of vinification such as applied techniques and processing conditions. Additional factors include the use of fining agents and the conditions under which the final product is stored [9,10,11,12,13]. The main sources of individual elements in wines are summarized in Table 1.

Different elements can influence wine characteristics, including aroma, color, and mouthfeel. Some metals are essential for yeast metabolism, whereas others can cause haze, browning, or instability. Table 2 summarizes the effects of selected elements on wine quality.

Numerous studies have applied multi-elemental analysis combined with chemometric tools to investigate geographical origin, varietal differentiation, and technological influences in wines. Selected representative studies are summarized in Table 3.

Wine adulteration is a growing concern in the global food market, as it can affect not only the authenticity and economic value of the product but also consumer safety. The most common types of wine adulteration include water dilution [20], the addition of sugars or other sweetening agents [21,22], the use of exogenous organic acids, alcohols, preservatives, or artificial flavor compounds [23,24], as well as blending with cheaper wines or fruit juices [25,26] and mislabeling related to grape variety or geographical origin [26,27,28]. In the case of white wines, one of the potential adulterants is apple juice, which can alter the chemical composition, color, and acidity of the beverage while remaining difficult to detect visually. Although the elemental composition of both red and white wines has been extensively investigated, only limited studies have focused on the adulteration of white wines with apple juice. Previous research mainly addressed this issue by determining chlorogenic acid content [29] or using electrochemical measurements [30]. In comparison with approaches based on a single chemical marker, multi-element ICP–OES analysis provides simultaneous information on a broad set of inorganic constituents and enables evaluation of coordinated compositional changes in the wine matrix. When combined with statistical and chemometric tools, multi-element ICP–OES data may provide complementary information on adulteration-related compositional changes and support the screening-oriented assessment of wine authenticity. Against this background, the present study combined multi-element ICP–OES analysis with statistical and chemometric methods to examine the response of Riesling wines to controlled apple juice addition. The aim was to characterize changes in their elemental composition and pH at three addition levels (5%, 10%, and 20% *v*/*v*) and to explore the resulting patterns within this model system.

**Table 3:** Overview of selected studies employing ICP-based techniques for wine analysis.

Wine Samples Analyzed	Grape Varieties/Origin	Elements Determined	Analytical Techniques	Sample Preparation	Main Findings/Conclusions	Ref
27 white wines from Romania	Sauvignon Blanc, Fetească Albă, Riesling Four main wine-producing regions of Romania	Al, As, Ba, Ca, Cd, Ce, Co, Cu, Cr, Cs, Fe, Hg, K, La, Mg, Mn, Mo, Na, Ni, Pb, Rb, Sr, Ti, Zn, V Isotopic ratios: ^206^Pb/^207^Pb, ^208^Pb/^206^Pb, ^86^Sr/^87^Sr	ICP–MS	Samples were mineralized and diluted (1:20, *v*/*v*). 2.5 mL of wine was mixed with 2.5 mL of ultrapure nitric acid.	Concentrations of toxic metals were below the limits established by the OIV. Among major elements, potassium showed the highest concentrations, followed by magnesium, calcium, and sodium. Comparison of isotopic ratios and trace element concentrations in wines and vineyard soils may be used for wine authentication.	[31]
57 monovarietal white wines from Argentina	Torrontés, Chardonnay, Sauvignon Blanc Four main wine-producing regions of Argentina	As, Ba, Be, Bi, Cd, Co, Cu, Ge, Hg, Li, Mn, Mo, Ni, Pb, Rb, Sr, Tl, V	ICP–MS	Samples were diluted (1:10) with 1% HNO_3_.	Wine samples were discriminated based on the concentrations of five ultra-trace elements (Ba, As, Pb, Mo, Co), which varied depending on the wine-producing region.	[27]
Five sample sets covering the entire production chain (from grape must to finished white wine)	Five German wine-growing regions: Rheingau, Rheinhessen, Baden, Pfalz, Mittelrhein	Al, Ag, As, B, Be, Ca, Cd, Co, Cr, Cu, Fe, Ga, Ge, Li, Mg, Mn, Mo, Nb, Ni, Pd, Rb, Rh, Ru, Sc, Se, Sn, Sr, Ti, Y, Zn, Zr, V	ICP–MS	Samples were filtered (0.45 µm cellulose acetate membrane). Digestion: 2.5 mL wine + 2.5 mL HNO_3_ (14 M). The digest was transferred to 25 mL volumetric flasks, resulting in a 1:10 dilution.	Concentrations of Li, B, Mg, Ca, Rb, Cs and Pb remained nearly constant throughout winemaking and were independent of bentonite addition. Concentrations of Al, Ba, Cu, Y and Zr decreased after fermentation but increased again after clarification. Co, Mn, Fe, Sr, Zn and V appeared to depend exclusively on the winemaking process.	[15]
23 white wines produced conventionally (12) and organically (11)	Italy	Al, As, B, Ca, Cd, Co, Cr, Cu, Fe, K, Li, Mg, Mn, Na, Ni, Pb, Sr, V, Zn	ICP–OES	Samples were filtered (0.45 µm nylon filter) and diluted. For trace elements, a 2.5-fold dilution was applied, while major elements were diluted 10-fold.	No significant differences in mineral composition were observed between conventional and organic wines, except for Ni, which showed higher concentrations in organic samples. Comparison with literature revealed a lack of consistency among published studies comparing conventional and organic wines.	[32]
180 wine samples: 79 red, 75 white and 26 rosé wines	23 countries (predominantly Poland; *n* = 42)	Group 1: Ag, B, Ba, Be, Bi, Cd, Co, Cr, Cu, Li, Mn, Mo, Ni, Pb, Rb, Sb, Sn, Sr, Te, Tl, U, Zn; Group 2: Ca, Fe, K, Mg, Ti; Group 3: Hg	Group 1: ICP–MS; Group 2: ICP–OES; Group 3: CVAAS	Sample digestion: 4 mL wine + 4 mL HNO_3_ (69%). The digest was transferred to 25 mL volumetric flasks and diluted with distilled water.	Red wines showed higher mean and median concentrations of B, Ba, Cr, Cu, Mn, Sr and Zn. White wines exhibited higher levels of Ag, Be, Bi, Cd, Co, Li, K and Ti. Rosé wines were characterized by higher Fe and U concentrations. Polish wines (*n* = 42) showed the lowest concentrations of Be, Mn and Zn.	[16]
60 wine samples: 26 red and 34 white wines	Romania (Valea Călugărească, Murfatlar) and Moldova	Cr, Ni, Rb, Sr, Ag, Zn, Mn, Cu, Co, V, Pb, Be	ICP–MS	Sample digestion: 2.5 mL wine + 2.5 mL concentrated nitric acid. The digest was transferred to 25 mL volumetric flasks and diluted with distilled water.	A correlation between the elemental composition of wines and vineyard soils was observed. Ag, Cr, Mn and Sr were identified as key discriminators between Romanian wine regions. Within individual regions, differentiation was based on Ag, Cr, Cu, Ni, Sr and Zn (Valea Călugărească); Mn, Rb and Zn (Murfatlar); and Co, Pb and V (Moldova).	[26]
53 Polish wine samples: 31 white wines and 22 red wines; 31 monovarietal wines, 18 multivarietal wines, and 4 fruit wines (cherry, pear, apple, blackcurrant)	Poland	Group 1: Al, As, B, Ba, Be, Cd, Co, Cr, Cu, Fe, Hg, Li, Mn, Ni, Pb, Sb, Se, Sr, Ti, V, Zn, Zr; Group 2: Ca, Na, K, Mg	Group 1: ICP–MS; Group 2: ICP–OES	Samples were diluted (1:10) with deionized water without any prior sample preparation.	White wines showed higher concentrations of Al, As, Be, Ca, Co, Cu, Fe, Hg, Li, Mg, Na, Pb, Sb, Ti, V, Zn and Zr. Red wines exhibited higher concentrations of Ba, Cd, Cr, K, Mn, Se and Sr. Health risk assessment indicators indicated that the analyzed samples do not pose a potential risk to consumer health.	[17]
90 white and red wine samples	Six regions of Greece; nine grape varieties; vintages 2017 and 2018	Na, Mg, P, K, Ca, Cu, Co, Cr, Zn, Sn, Fe, Mn, Li, Be, B, V, Sr, Ba, Al, Ag, Ni, As, Hg, Pb, Sb, Cd, Ti, Ga, Zr, Nb, Pd, Te, La, Sm, Ho, Tm, Yb, W, Os, Au, Tl, Th, U	ICP–MS	Sample digestion: 2.5 mL wine + 5 mL HNO_3_. The digest was transferred to vials and diluted with distilled water to a final volume of 20 mL.	Seasonal variations between vintages significantly affected elemental concentrations, with higher levels observed in wines from the 2018 vintage compared to 2017. The samples showed good classification based on elemental composition when grape varieties were considered.	[33]
24 white wines	Moravia region (Czech Republic); grape varieties: Grüner Veltliner, Rhine Riesling, Müller-Thurgau	Group 1: Li, Be, Cd, Sn, Sb, Ba, La, Ce, Pr, Nd, Sm, Eu, Gd, Tb, Dy, Ho, Er, Tm, Yb, Lu, Pb, Bi, V, Cr, Co, Ni, Cu, As, Y, Mo; Group 2: Al, Fe, Mg, Mn, Sr, Zn, Ca, K, Na	Group 1: ICP–MS; Group 2: ICP–OES	Samples were diluted 1:1 with deionized water and filtered immediately prior to analysis using a 0.45 µm membrane.	The investigated grape varieties differed mainly in the mean concentrations of rare earth elements and their ratios. Principal component analysis (PCA) separated the samples into varietal groups with satisfactory performance.	[34]
111 sparkling wines	Four countries: Brazil (41), Argentina (42), Spain (11), France (17)	12 elements: Al, B, Ba, Ca, Cu, Fe, K, Li, Mg, Mn, Na, Sr	ICP–OES	Undiluted samples were analyzed for Al, B, Ba, Cu, Fe, Li, Mn and Sr. For Ca, K, Mg and Na determination, samples were diluted 1:10 (*v*/*v*) with 1 mol/L HNO_3_. After bottle opening, wines were degassed using an ultrasonic bath.	Using B, K and Na concentrations, wine samples were classified with 94% accuracy. Ca, K and Na enabled discrimination between South American and European wines. Brazilian and Argentinian wines were differentiated based on Mn concentrations, while Spanish and French wines were separated using the B/K ratio.	[28]
272 Slovenian wines: 193 white and 79 red wines	Slovenia	Group 1: Li, Be, Sc, Ti, V, Cr, Co, Ni, Ga, Ge, As, Se, Y, Zr, Nb, Mo, Ru, Rh, Pd, Ag, Sn, Sb, Ba, La, Ce, Nd, Gd, Dy, Ta, W, Re, Os, Ir, Pt, Au, Hg, Tl, Pb, Bi; Group 2: Al, B, Ca, Cu, Fe, K, Mg, Mn, Na, Y, Zn	Group 1: ICP–MS; Group 2: ICP–OES	Samples were diluted using 2% (*v*/*v*) nitric acid: ten-fold for ICP–MS and three-fold for ICP–OES.	Both white and red wines could be grouped into subregions. While PCA did not provide satisfactory classification, counter-propagation artificial neural networks (CPANN) successfully grouped samples according to region.	[35]
45 Czech wines	Moravia region; conventional (35) and organic (10) production	K, Na, Mg, Ca, Cr, Zn, Pb, Cd, Co, Mn, Fe, Cu, Ni, Al, P	ICP–OES	Samples were filtered using 0.45 µm nylon syringe filters.	Czech wines did not differ from wines from Europe, Australia or South America in terms of Mg, Ca, K, P, Zn, Pb, Cd, Co, Mn, Fe, Cu, Ni and Al concentrations. All analyzed wines were within the limits established by the OIV.	[36]

## 2. Result and Discussion

### 2.1. Elemental Profile of Authentic White Wines

To evaluate whether the Riesling wines selected for the adulteration experiment differed from the broader background of authentic white wines, their elemental profile was compared with that of the remaining white wine samples using the Mann–Whitney U test. After Holm correction for multiple comparisons, significant differences were observed for Ca, Cu, K, Ni, and Zn (Table 4). These elements were generally higher in Riesling wines than in the remaining white wines, indicating that the Riesling samples exhibited some variety-related elemental characteristics. Therefore, the subsequent adulteration experiment should be interpreted as a controlled Riesling-based model, while the remaining white wines provide a reference background for the natural elemental variability of authentic white wines.

The higher concentrations of Ca, Cu, K, Ni, and Zn observed in Riesling wines should not be interpreted solely as a varietal effect, because the Riesling group consisted of wines from Germany, whereas the remaining white wines represented different grape varieties and countries of origin. Previous studies have shown that the elemental profile of wine is influenced by multiple overlapping factors, including wine type, geographical origin, grape variety, vineyard soil, local environmental conditions, and winemaking practices [13,14,15,16,17]. In particular, Ca and K may reflect differences related to grape mineral composition, soil properties, climatic conditions, and technological factors [15,16,17]. Higher Cu and Zn levels may reflect both vineyard-related and technological sources, including soil composition, agrochemical inputs, and winemaking or storage conditions [13,14,17,37]. Therefore, the differences observed between Riesling and the remaining white wines most likely reflect the combined influence of varietal, geographical, soil-related, and technological factors rather than a purely varietal effect.

### 2.2. Effect of Apple Juice Addition on Riesling Wines

In the ICP–OES analysis, 60 white wine samples and 45 Riesling wine samples intentionally adulterated with apple juice, as well as the apple juice used for adulteration, were analyzed for the content of 24 elements: silver (Ag), aluminum (Al), barium (Ba), beryllium (Be), calcium (Ca), cadmium (Cd), cobalt (Co), chromium (Cr), copper (Cu), iron (Fe), potassium (K), magnesium (Mg), manganese (Mn), sodium (Na), nickel (Ni), phosphorus (P), lead (Pb), sulfur (S), antimony (Sb), tin (Sn), strontium (Sr), titanium (Ti), vanadium (V), and zinc (Zn). In all analyzed samples, the concentrations of Ag, Be, Co, Cd, and Sb were below the limit of quantification (LOQ; see Section 3.2.1) and therefore were excluded from further statistical evaluation.

To provide an overview of the effect of apple juice addition on the elemental composition of Riesling wines, the changes in elemental concentrations between unadulterated and adulterated samples are summarized in Table 5. The table presents the direction of changes observed after apple juice addition, together with the corresponding statistical significance. This approach allows a clear identification of elements whose concentrations decreased or increased in response to adulteration.

**Table 4:** Comparison of elemental concentrations between unadulterated Riesling wines and other authentic white wines.

Element	Al	Ba	Ca	Cr	Cu	Fe	K	Mg	Mn	Na	Ni	P	Pb	S	Sn	Sr	Ti	V	Zn
Riesling, mean ± SD (mg/L)	8.028 ± 0.929	0.182 ± 0.015	104.5 ± 2.414	0.020 ± 0.003	0.260 ± 0.152	1.786 ± 0.421	771.9 ± 24.575	83.53 ± 3.933	1.174 ± 0.188	32.58 ± 4.590	0.024 ± 0.0022	176.886 ± 38.453	0.024 ± 0.0059	156.0 ± 5.074	0.182 ± 0.017	0.524 ± 0.138	0.040 ± 0.013	0.016 ± 0.003	1.097 ± 0.189
Riesling, median (min–max) (mg/L)	7.815 (6.267–9.408)	0.181 (0.157–0.211)	104.5 (100.5–109.2)	0.017 (0.017–0.024)	0.322 (0.078–0.408)	1.962 (1.207–2.431)	782.4 (734.5–812.2)	82.49 (78.44–90.73)	1.093 (0.921–1.404)	34.51 (24.49–37.58)	0.025 (0.018–0.025)	158.2 (154.7–252.5)	0.023 (0.016–0.035)	153.6 (150.4–164.2)	0.181 (0.151–0.209)	0.419 (0.409–0.691)	0.035 (0.028–0.078)	0.019 (0.013–0.019)	1.040 (0.913–1.606)
Other white wines, mean ± SD (mg/L)	7.577 ± 1.279	0.173 ± 0.044	81.94 ± 16.32	0.017 ± 0.005	0.098 ± 0.103	1.233 ± 0.794	667.9 ± 167.4	79.38 ± 17.80	0.901 ± 0.410	47.30 ± 17.64	0.019 ± 0.017	197.2 ± 58.42	0.020 ± 0.013	150.1 ± 24.23	0.186 ± 0.034	0.639 ± 0.372	0.034 ± 0.014	0.024 ± 0.054	0.649 ± 0.448
Other white wines, median (min–max) (mg/L)	7.600 (5.193–10.38)	0.171 (0.103–0.250)	81.64 (46.81–114.6)	0.017 (0.011–0.030)	0.039 (0.002–0.317)	0.889 (0.234–3.549)	683.5 (408.6–1131)	76.12 (34.08–108.8)	0.820 (0.274–1.640)	51.16 (20.73–79.84)	0.018 (0.005–0.057)	196.4 (102.0–320.8)	0.017 (0.004–0.066)	147.2 (98.09–202.8)	0.179 (0.130–0.256)	0.582 (0.195–1.538)	0.028 (0.016–0.072)	0.006 (0.001–0.219)	0.627 (0.027–1.669)
*p*-value	0.172	0.267	6.20 × 10^−7^	0.055	9.25 × 10^−5^	0.009	9.25 × 10^−5^	0.082	0.013	0.037	0.000	0.194	0.023	0.112	0.878	0.772	0.025	0.013	0.000
Holm-adjusted *p*-value	0.860	0.860	1.18 × 10^−5^	0.438	0.002	0.126	0.002	0.571	0.173	0.335	0.004	0.860	0.249	0.674	1.000	1.000	0.253	0.173	0.002
Significant after Holm	NO	NO	YES	NO	YES	NO	YES	NO	NO	NO	YES	NO	NO	NO	NO	NO	NO	NO	YES

**Table 5:** Basic statistics of elemental concentrations determined by ICP–OES in Riesling wine samples (*n* = 15), Riesling wine samples adulterated with apple juice (*n* = 45; 5%, 10%, and 20% *v*/*v*), and apple juice used for adulteration (*n* = 3). Values are expressed in mg/L.

	Al	Ba	Ca	Cr	Cu	Fe	K	Mg	Mn	Na	Ni	P	Pb	S	Sn	Sr	Ti	V	Zn
Wine samples of Riesling variety (*n* = 15)																			
Mean	8.028	0.182	104.5	0.020	0.259	1.786	771.9	83.53	1.174	32.58	0.024	176.9	0.024	156.0	0.182	0.524	0.040	0.016	1.097
Median	7.815	0.180	104.5	0.017	0.322	1.962	782.4	82.49	1.093	34.51	0.024	158.2	0.023	153.6	0.181	0.419	0.035	0.019	1.040
Min	6.267	0.157	100.5	0.017	0.078	1.207	734.6	78.44	0.920	24.49	0.018	154.6	0.016	150.4	0.151	0.409	0.028	0.013	0.913
Max	9.408	0.211	109.2	0.024	0.408	2.431	812.2	90.73	1.404	37.58	0.025	252.5	0.035	164.2	0.209	0.691	0.078	0.019	1.606
SD	0.929	0.015	2.414	0.003	0.152	0.421	24.58	3.933	0.188	4.590	0.002	38.45	0.006	5.074	0.017	0.138	0.013	0.003	0.189
Wine samples of Riesling variety with addition of apple juice (*n* = 45)																			
Mean	6.115	0.170	88.75	0.021	0.219	1.485	747.9	71.08	1.048	37.00	0.031	137.0	0.069	130.7	0.137	0.484	0.027	0.013	1.007
Median	5.884	0.169	88.24	0.022	0.263	1.552	746.1	70.38	1.028	35.51	0.019	130.2	0.023	133.4	0.133	0.407	0.028	0.013	1.018
Min	4.553	0.144	74.73	0.016	0.068	1.124	683.8	59.76	0.772	27.58	0.012	109.8	0.010	109.8	0.100	0.346	0.022	0.006	0.777
Max	8.074	0.216	100.4	0.036	0.507	1.892	803.5	82.57	1.316	59.77	0.456	185.6	0.867	145.4	0.180	0.672	0.041	0.020	1.472
SD	0.923	0.017	6.353	0.005	0.127	0.230	32.83	6.593	0.170	8.041	0.065	20.27	0.180	11.11	0.022	0.119	0.005	0.002	0.137
Apple juice (*n* = 3)																			
Mean	5.954	0.217	37.40	0.017	0.043	0.501	966.0	33.45	0.341	101.3	0.006	67.84	0.010	19.17	0.139	0.186	0.003	0.001	0.611
Median	5.781	0.215	37.56	0.017	0.045	0.502	964.9	33.61	0.339	101.3	0.006	67.96	0.010	19.22	0.135	0.189	0.003	0.001	0.611
Min	4.864	0.203	36.37	0.017	0.039	0.475	956.0	33.09	0.339	97.49	0.006	67.39	0.010	18.89	0.117	0.178	0.003	0.001	0.611
Max	7.219	0.234	38.28	0.017	0.045	0.526	977.2	33.65	0.344	105.2	0.006	68.17	0.010	19.40	0.166	0.190	0.003	0.001	0.611
SD	1.187	0.015	0.966	0.000	0.004	0.025	10.66	0.313	0.003	3.845	0.000	0.401	0.000	0.256	0.025	0.007	0.000	0.000	0.000

The generally lower concentrations of most elements in apple juice compared to white wine samples can be attributed to differences in the raw material, production technology, and absence of fermentation processes, which in wines contribute to the enrichment of the matrix with microelements originating from yeast activity, fermentation lees, and contact with winemaking equipment [38]. The higher sodium (Na) content observed in the analyzed apple juice can be explained by both natural and technological factors. Apples are often cultivated in soils with increased salinity [39]. Moreover, apple juices are frequently produced by reconstituting concentrated juice with process water, which is sometimes softened using sodium chloride (NaCl), thereby increasing the final sodium concentration in the product. In addition, the juice industry commonly employs preservative or antioxidant additives in the form of sodium salts, such as sodium benzoate (E211) and sodium ascorbate (E301), which also contribute to the overall Na^+^ content [40]. Consequently, although the total elemental content in apple juices is generally lower than in white wines, the sodium concentration may be relatively higher due to the influence of the raw material, process water, and technological additives. It should be noted that the elemental composition of apple juice may vary depending on apple cultivar, geographical origin, growing conditions, and processing technology, as previously observed for apple-based beverages [41]. Therefore, the findings of the present study should be interpreted in relation to the specific commercial clarified apple juice used in this model experiment.

To provide a general comparison between unadulterated wine samples and wine samples adulterated with apple juice, the Wilcoxon signed-rank test for paired samples was applied (Table 6). The choice of this test was based on the experimental design, as the compared samples did not represent independent groups. Each Riesling sample was analysed both in its unadulterated form and after the addition of apple juice. For each sample and each element, the value obtained for the L0 level was used to represent the unadulterated wine, whereas the mean value calculated from the L5, L10, and L20 levels was used to represent the adulterated wine. This approach allowed the comparison to account for changes occurring within the same samples, rather than treating the different adulteration levels as independent observations.

After applying the Holm correction, statistically significant differences between unadulterated wines and wines adulterated with apple juice were observed for 14 out of 19 analysed elements.

The effect of apple juice addition level on elemental concentrations in Riesling wine samples was evaluated using Friedman’s test. When significant differences were found, post hoc Wilcoxon signed-rank tests with Holm correction were performed. The results are summarized in Table 7, which presents the median concentrations at L0, L5, L10, and L20; the *p*-values obtained from Friedman’s test; and the significant pairwise differences between adulteration levels (* pHolm < 0.05, ** pHolm < 0.01, *** pHolm < 0.001). Detailed Holm-adjusted *p*-values obtained from post hoc Wilcoxon signed-rank tests for all pairwise comparisons are provided in Table S1 (Supplementary Materials).

For most of the analyzed elements, the level of apple juice addition had a significant effect on their concentrations. The clearest gradual decrease with increasing adulteration level was observed for Ca, Mg, Mn, P, S, and Sr. For these elements, all pairwise comparisons remained statistically significant after Holm correction, indicating a strong relationship between the proportion of apple juice added and the decrease in their concentrations in the wine matrix.

For some elements, such as Al, Sn, and Ti, significant differences were mainly observed between unadulterated samples and samples containing apple juice, whereas further increases in the apple juice proportion did not result in significant differences among the L5, L10, and L20 levels. The concentrations of these elements were lower in the apple juice than in the unadulterated Riesling wines, and therefore the initial decrease after apple juice addition is consistent with a dilution-related effect. However, the absence of a clear concentration-dependent trend indicates that Al, Sn, and Ti are less suitable as indicators of adulteration level than elements showing a consistent gradual response, such as Ca, Mg, Mn, P, S, and Sr. A more complex pattern was observed for Ba, K, Na, and Zn, for which selected pairwise differences were significant, but the changes did not follow a simple monotonic trend. In the case of Ni, Pb, and V, Friedman’s test did not indicate significant differences among the adulteration levels. As some pairwise differences were significant only before adjustment, the final interpretation was based exclusively on Holm-adjusted *p*-values.

Although Ca, K, Mg, Mn, P, and S decreased in all 15 paired comparisons between L0 and L5, the 5% (*v*/*v*) addition level should not be interpreted as a detection limit for adulteration in unknown wines, as lower addition levels and classification performance were not evaluated. The Riesling samples originated from two German wine-producing regions, Mosel (*n* = 6) and Palatinate (*n* = 9), and the decreases observed between L0 and L5 occurred in samples from both regions. Nevertheless, all Riesling samples were from the 2023 vintage, and the experimental design did not allow the independent effects of region, vintage, or winemaking practices to be determined. In addition, further studies involving other fruit juices would be needed to evaluate the specificity of the observed elemental pattern.

**Figure 1:**
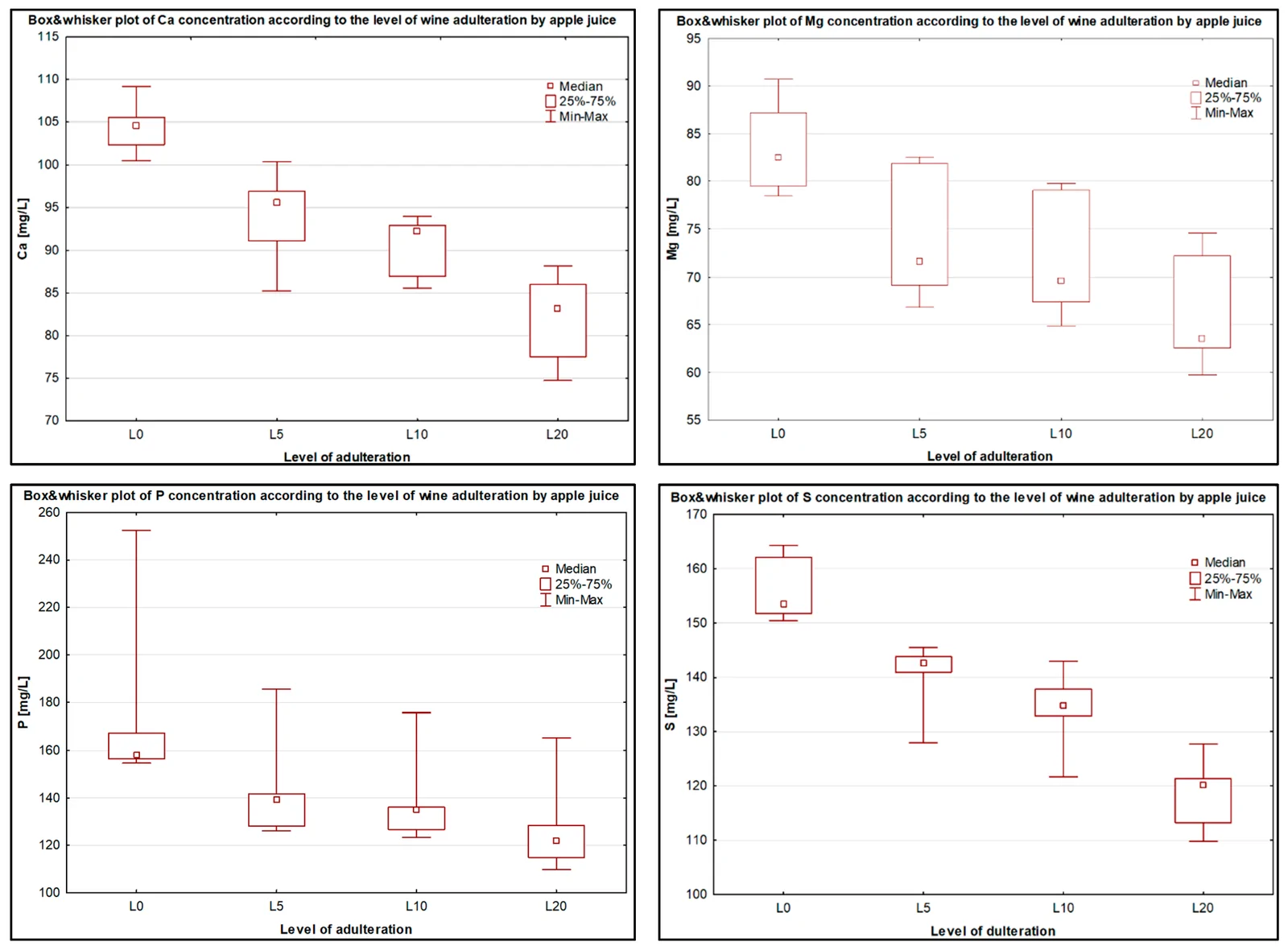
Box-and-whisker plots of calcium (Ca), magnesium (Mg), phosphorus (P) ad sulphur (S) concentrations in the Riesling wine samples at different levels of apple juice adulteration (L0–L20).

To visualize the distribution of selected elemental concentrations across the individual adulteration levels, box-and-whisker plots were prepared for elements showing the most evident and consistent changes, namely Ca, Mg, P, and S (Figure 1). These plots correspond to the L0, L5, L10, and L20 groups summarized in Table 7. These elements were chosen as representative examples because their concentrations showed clear downward shifts with increasing levels of apple juice addition. The use of box-and-whisker plots enables visualization not only of median changes, but also of the variability within each adulteration level. Overall, the graphical presentation shows a pronounced decrease in Ca, Mg, P, and S concentrations from unadulterated wines to samples containing increasing proportions of apple juice, supporting the interpretation of a dilution effect caused by the addition of apple juice.

Pearson correlation analysis was used to evaluate the relationships among the determined elements and to verify whether elements showing similar changes after apple juice addition formed common correlation groups. The full correlation matrix is presented in Table S2 in the Supplementary Materials.

The correlation matrix showed strong positive relationships between selected elements whose concentrations decreased with increasing adulteration level. Particularly high correlation coefficients were obtained for Mg–Mn (r = 0.861), Ca–S (r = 0.805), Mg–S (r = 0.762), and Ca–Mg (r = 0.761). These relationships indicate similar behaviour of these elements in Riesling wine samples and support the interpretation that their decrease was mainly associated with the dilution of the wine matrix caused by apple juice addition.

A different behaviour was observed for sodium. In contrast to most elements, whose concentrations decreased after apple juice addition, Na increased, particularly at the highest adulteration level. This was reflected in negative correlations between Na and elements representing the dilution-related trend, including Ca (r = −0.607), Mg (r = −0.588), S (r = −0.609), and Mn (r = −0.492). This correlation pattern indicates that sodium did not follow the typical decreasing trend observed for the main mineral components of wine, but behaved differently, which is consistent with its higher concentration in apple juice.

A very strong positive correlation was also observed between Al and Sn (r = 0.982), together with positive relationships between these elements and Ti. This may indicate similar behaviour of these elements in the analyzed system; however, their interpretation should be made with caution due to their low concentration levels and the possible influence of matrix-related or analytical factors. Strong positive correlations were also found for Cu–Fe (r = 0.856) and Cu–Sr (r = 0.823), suggesting that some elements may be associated not only with the dilution effect, but also with technological factors or the specific characteristics of the wine matrix.

PCA performed using all determined elements showed only partial separation of Riesling wine samples according to the level of apple juice addition (Figure S1, Supplementary Materials). In this model, the first two principal components explained 50.46% of the total variance, with PC1 accounting for 28.68% and PC2 for 21.78%. Although unadulterated samples were generally shifted towards positive PC1 values and samples with the highest apple juice addition were more frequently located at negative PC1 values, a considerable overlap between the adulteration levels was observed. This suggests that the inclusion of all elements, including variables with weak or non-systematic responses to apple juice addition, obscured the structure related to adulteration level in the PCA score plot.

**Figure 2:**
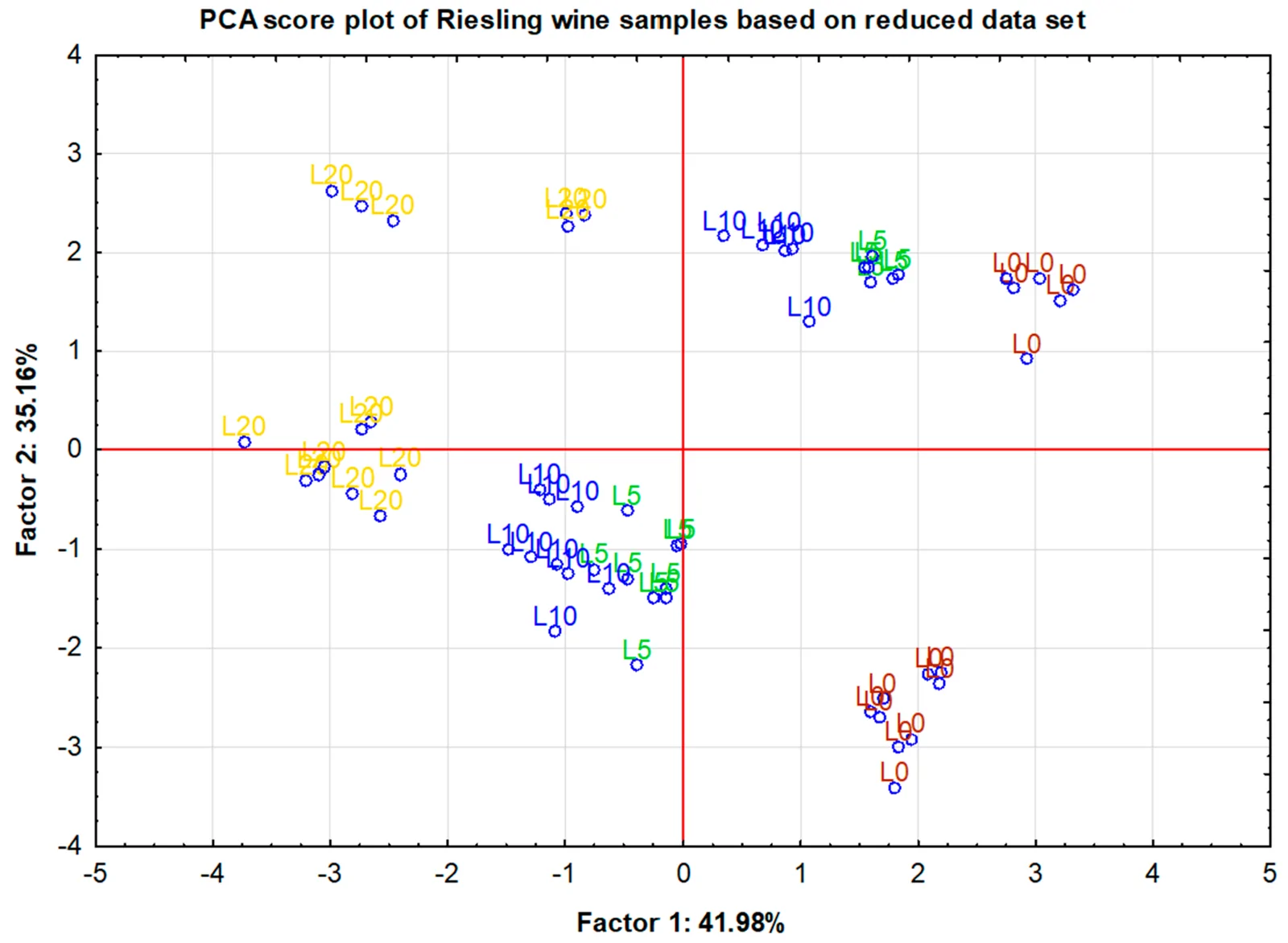
PCA score plot showing the projection of Riesling wine samples (*n* = 60) onto the factor plane based on selected diagnostic elements: Ca, Mg, Mn, Na, P, S, and Sr.

Therefore, a reduced PCA model was constructed using selected elements: Ca, Cu, Fe, Mg, Mn, Na, P, Sr, and S. The selection was based on statistically significant changes across adulteration levels, consistent concentration trends, and correlation patterns associated with apple juice addition. The reduced data set included elements representing the main dilution-related pattern, such as Ca, Mg, Mn, P, S, and Sr, together with Na, which exhibited an opposite behaviour, and Cu and Fe, which showed significant changes and were associated with matrix- or technology-related variation. PCA performed on the reduced elemental data set yielded three components with eigenvalues greater than 1 according to the Kaiser criterion, explaining 92.55% of the total variance (Table S3, Supplementary Materials). For visualization and interpretation purposes, the first two principal components were presented in Figure 2, as they accounted for 77.14% of the total variance, with PC1 explaining 41.98% and PC2 explaining 35.16%.

In the reduced PCA model, samples with the highest adulteration level (20% *v*/*v*) were clearly shifted towards negative PC1 values, whereas unadulterated samples were mainly located on the positive side of PC1. Samples adulterated at lower levels, especially 5% and 10% (*v*/*v*), occupied intermediate positions and partially overlapped with unadulterated wines. This suggests that low levels of apple juice addition caused more subtle changes in the elemental profile, which may be partly masked by the natural variability of the wine samples. In contrast, the more pronounced shift in the 20% samples along PC1 indicates a stronger change in their elemental composition. Overall, the reduced PCA model provided a clearer visualization of the changes associated with apple juice addition than the model based on the full elemental data set.

The factor loadings of the reduced PCA model (Table S4, Supplementary Materials) supported the interpretation of the sample distribution observed in the score plot. PC1 was mainly associated with elements showing dilution-related behaviour, with high positive loadings for Mg (0.936), Ca (0.902), S (0.849), and Mn (0.698), whereas Na showed a strong negative loading (−0.764). This indicates that PC1 reflected the main contrast between elements decreasing with apple juice addition and sodium, which exhibited an opposite trend. PC2 was primarily characterized by strong negative loadings for Cu (−0.962), Fe (−0.953), and Sr (−0.773), suggesting the contribution of additional matrix- or technology-related variability among Riesling samples. PC3 was mainly associated with P (0.799) and, to a lesser extent, Sr (−0.622), but it was not used for graphical interpretation in the score plot. Overall, the loading structure of the reduced PCA model was consistent with the concentration trends observed for the selected elements.

### 2.3. pH Analysis

The effect of apple juice addition on the pH of wines was evaluated by measuring the pH of adulterated samples. For Riesling wines, three levels of apple juice adulteration were examined: 5%, 10%, and 20% (*v*/*v*). The obtained results for pure wines and for samples with the respective levels of adulteration are summarized in Table 8.

The results obtained in this study indicate an increase in the pH of Riesling wines following the addition of apple juice, which can be directly attributed to the difference in acidity between these two products. The pH values determined for the pure Riesling wines ranged narrowly between 3.08 and 3.13, which is consistent with literature data for white wines. According to the previous studies, the pH of white wines usually falls within the range of 3.0–3.3 (the average pH of white wines ~3.01), while red wines exhibit slightly higher values (around 3.33) due to malolactic fermentation, which reduces their overall acidity [16].

The pH value of the apple juice analyzed in this work was 3.57, which also agrees with the recent literature data. Reported pH values for apple juices typically range from 3.04 to 3.69, and in some commercial varieties may reach up to 4.0 [42,43]. Cai et al. (2020) reported a pH of 3.53 ± 0.01 for freshly pressed apple juice, with values remaining stable between 3.50 and 3.57 after various filtration and storage treatments [44]. Similar findings were presented by Gajek et al. (2021), who compared the physicochemical properties of several apple juices and the ciders produced from them, obtaining median pH of 3.47 [41].

The difference in pH between the Riesling wines and the apple juice used for adulteration fully explains the observed increase in pH in the adulterated samples, particularly at the highest level of addition (20% *v*/*v*). This behaviour is entirely consistent with expectations, as the incorporation of a less acidic component such as apple juice leads to a reduction in the overall acidity of the mixture and a corresponding increase in pH.

## 3. Material and Methods

### 3.1. Samples

The study was conducted on 60 white wines representing seven grape varieties: Riesling, Chardonnay, Sauvignon Blanc, Gewürztraminer, Solaris, Soave, and Moscato. The samples originated from nine countries—Germany, Poland, Italy, Hungary, Australia, Moldova, Chile, the United States, and Spain. The analyzed wines differed in their residual sugar content, ranging from dry to sweet styles. All wines were commercially available and purchased from retail stores in Poland. Wine samples were collected immediately after bottle opening and stored under refrigeration at 4 °C until analysis. A brief description of the analyzed samples is provided in Table 9. Detailed information for each individual sample, including grape variety, country and region of origin, vintage, alcohol content, and residual sugar category, is provided in Table S5 in the Supplementary Materials.

The full set of authentic white wines was used to provide a reference background for the natural elemental variability of white wines and to compare the elemental profile of Riesling wines with that of the remaining wine samples. However, the intentional adulteration experiment was performed only on Riesling wines, which were selected as a controlled wine matrix. The selection of the adulterant was guided by literature data [30]. Intentional adulteration of wines was performed using commercially available, 100% clarified apple juice. Fifteen samples of Riesling wine were chosen, and measured volumes of apple juice were added to each to achieve three adulteration levels of 5, 10, and 20% (*v*/*v*) (a total of 45 samples were obtained).

The apple juice used for adulteration was a commercially available 100% clarified apple juice purchased from a retail store in Poland. According to the product information, the juice was produced from a mixture of different commonly available cultivated apple varieties originating from Poland. The same juice batch was used for all adulteration experiments. The elemental composition of the juice was determined using the same ICP–OES procedure as for the wine samples and is presented in Table 5.

### 3.2. Research Procedures

#### 3.2.1. ICP-OES Measurement

**Figure 3:**
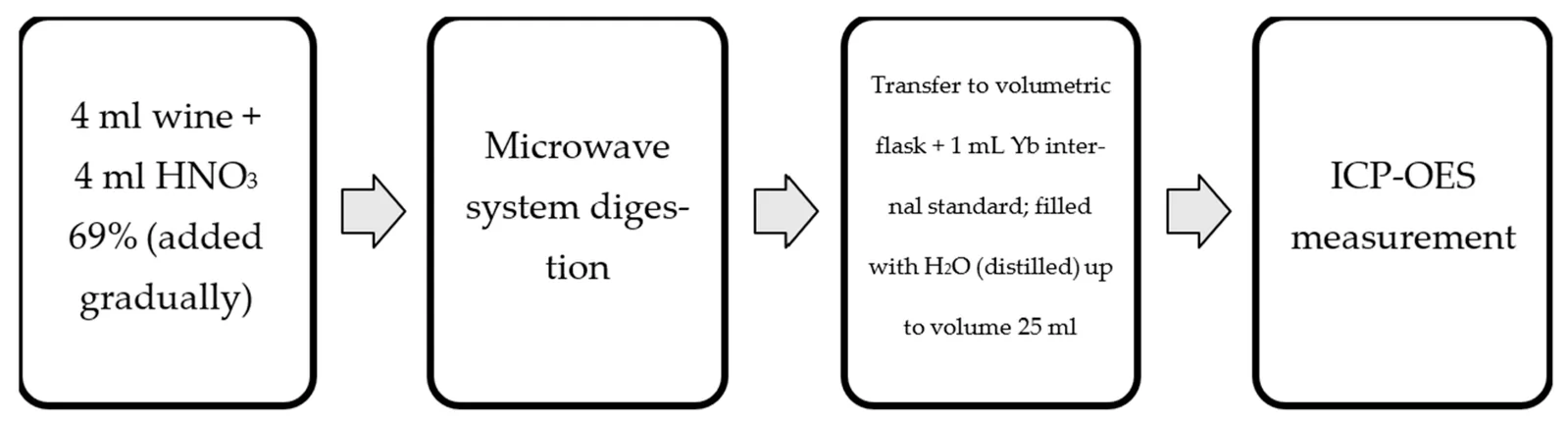
Procedure of sample preparation.

A crucial step preceding ICP analysis involved the mineralization of samples using an Ultrawave system (Milestone, Via Fatebenefratelli, Sorisole, Italy), carried out following the scheme depicted in Figure 3. The analytical procedure applied in this study was adapted from the methodology previously reported by Gajek et al. (2021) [16].

Determination of elemental content was carried out using Inductively Coupled Plasma Optical Emission Spectrometry (ICP-OES; Thermo Scientific, iCAP 7000 Series, Waltham, MA, USA). Measurements of Ca, K, Mg, and Na were performed in radial plasma configuration, whereas the remaining elements (Ag, Al, Ba, Cd, Co, Cr, Cu, Fe, Mn, Mo, Ni, P, Pb, S, Sb, Sn, Sr, Ti, V, Zn) were determined using an axial plasma view. Each wine sample was analyzed in triplicate. Signal reproducibility and instrumental stability were monitored through the Yb internal standard line. Argon served as the plasma and carrier gas, while a quartz torch and concentric nebulizer were employed.

Due to the fact that the certified wine material (THX012RM, Fera Science Ltd., Sand Hutton, York, UK) was limited to only four elements (Cd, Cu, Fr, Pb), the accuracy of the analytical method was further evaluated using TMDA-64 (fortified lake water, National Water Research Institute, Burlington, ON, Canada) characterized by a broad elemental profile, and the human hair Certified Reference Material NCS ZC 81002b (China National Analysis Center for Iron & Steel, Beijing, China), which was applied to assess elements not included in the wine material. All analytes exhibited very good linearity, with regression coefficients ranging from 0.998 to 1.000. The sensitivity of the developed method was expressed as limits of detection (LOD) and quantification (LOQ), calculated from the variability of blank samples following the relationships LOD = mean × 3SD and LOQ = 3 × LOD [45]. The resulting values are presented in Table 10.

The preparation and analytical procedure for the wine samples containing apple juice followed the same protocol as that used for the non-adulterated wine samples.

#### 3.2.2. pH Measurement

The pH of the analyzed wine samples was determined using a Basic 20+ pH meter (Crison Instruments S.A., Barcelona, Spain) equipped with a magnetic stirrer and automatic temperature compensation. Measurements were performed with a combined electrode incorporating both a glass and a silver chloride reference electrode in a single probe. Prior to analysis, the instrument was calibrated daily with standard buffer solutions of pH 4.01, 7.00, and 9.21 (Hach Company, Düsseldorf, Germany). The pH of each wine sample was measured within the same analytical session, and all determinations were carried out in triplicate. The reported pH values represent the arithmetic mean of the three independent measurements.

#### 3.2.3. Data Analysis

Statistical and chemometric analyses were performed using STATISTICA software, version 12.5 (New York, NY, USA). Descriptive statistics, including mean, median, minimum, maximum, and standard deviation, were calculated for the analyzed groups. Elements with concentrations below the limit of quantification in all examined samples were excluded from further statistical evaluation.

Prior to statistical testing, the normality of the data distribution was assessed using the Shapiro–Wilk test at a significance level of 0.05. Since the normality assumption was not met for most variables, nonparametric statistical tests were applied. To evaluate whether the Riesling wines selected for the adulteration experiment differed from the broader background of authentic white wines, elemental concentrations in unadulterated Riesling samples were compared with those in the remaining authentic white wine samples using the Mann–Whitney U test for independent groups.

The effect of apple juice addition on the elemental profile of Riesling wines was assessed using tests for dependent samples, as each wine sample was analyzed in its unadulterated form and after the addition of apple juice. For the general comparison between unadulterated and adulterated Riesling wines, the Wilcoxon signed-rank test was applied. In this analysis, the L0 sample represented the unadulterated wine, whereas the mean value calculated from the L5, L10, and L20 levels represented the adulterated variant of the same wine sample. To evaluate the influence of the adulteration level, Friedman’s test was used to compare elemental concentrations across four related groups: L0, L5, L10, and L20.

Since statistical analyses were performed separately for 19 elements, the Holm correction was applied to adjust for multiple comparisons. This correction was used to reduce the risk of false-positive results associated with multiple statistical testing. Differences were considered statistically significant when the Holm-adjusted *p*-value was below 0.05.

Pearson correlation analysis was performed to evaluate relationships among the determined elements in Riesling wine samples across all adulteration levels. Principal component analysis (PCA) was used as an unsupervised chemometric tool to visualize the structure of the data set, evaluate sample distribution, and identify variables contributing to the observed patterns. Principal component analysis (PCA) was performed on standardized data using the correlation matrix to visualize sample distribution and identify variables contributing to the observed patterns. PCA was first carried out using all determined elements, followed by a reduced model based on selected diagnostic elements. These variables were selected according to their statistical significance, concentration trends, and correlation patterns. Components with eigenvalues greater than 1 were retained according to the Kaiser criterion, and factor loadings were used for interpretation.

## 4. Conclusions

The present study showed that controlled addition of apple juice caused measurable changes in the multi-elemental profile of Riesling wines. The most consistent response was a gradual decrease in Ca, Mg, Mn, P, S, and Sr, consistent with a dilution-related change resulting from the lower concentrations of these elements in the apple juice used in this experiment. In contrast, Na showed a different behaviour, with a marked increase particularly at the 20% (*v*/*v*) addition level, consistent with its higher concentration in the apple juice matrix.

The statistical analyses identified changes across the investigated addition levels. In the paired comparison between L0 and L5, Ca, K, Mg, Mn, P, and S decreased in all 15 Riesling samples. The reduced PCA model provided a clearer visualization of compositional changes than the model based on all determined elements, particularly through the shift in samples containing 20% (*v*/*v*) apple juice along PC1. Samples at the lower addition levels partially overlapped with unadulterated wines.

Alongside the elemental changes revealed by ICP–OES, pH increased with increasing apple juice addition, providing complementary physicochemical information on the studied Riesling–apple juice system. In an assessment of wine authenticity, these measurements should be considered alongside other analytical indicators, such as organic-acid and sugar profiles, since decreases in elemental concentrations alone do not identify the added liquid. Comparisons involving other wines and juice sources, water and other fruit-juice additions, and lower addition levels would help assess how broadly the observed patterns apply and whether they can distinguish apple juice addition from other changes to wine.

## Figures and Tables

**Table 1:** Sources of selected elements in wine [14,15,16,17,18,19].

Element	Possible Sources in Wine	Reference
K	Naturally present in grapes; may indicate grape variety, soil, climate, or storage conditions	[16,17,18,19]
Cu	From soil and plant protection agents (copper sulfate, fungicides); may also originate from production equipment	[14,17]
Fe	Related to soil composition and maceration time; reflects Fe content in grapes	[14,16,17,19]
Zn	From soil and fertilizers, or contamination during processing and storage	[14,16,17,19]
Al	From production processes, e.g., bentonite used for clarification	[14,16,17,19]
Cr	From production equipment and chromium oxides used in processing	[14,17]
Pb	From pesticides, soil contamination, or corrosion of winemaking equipment	[14,15,17]
Ti	Indicator of soil type or bottle color additives	[14,17]
Ba	From soil and geological background	[14]
Mn	From soil; involved in enzymatic activity during fermentation	[14,16]
Cd	Anthropogenic contamination (fertilizers, pollution)	[14]
Ni	From stainless steel equipment	[14,16]

**Table 2:** Examples of elements present in wines and their impact on this product [14,17,19].

Element	Effect on Wine	Reference
Fe	Causes browning, haze, and metallic taste; activates metalloenzymes during fermentation	[14,17,19]
Cu	Induces haze and brownish precipitates in white wines; may increase astringency; supports yeast metabolism	[14,19]
Zn	Contributes to haze and browning; promotes yeast enzyme activity	[19]
Cr	May lead to browning and instability	[17,19]
Al	Causes browning and haze formation	[14,17,19]
Mn	Participates in oxidation reactions and enzyme activation	[14,19]
K	Affects acidity; high concentrations may cause instability	[16]
Pb	Induces instability and browning	[16]

**Table 6:** Statistically significant differences in elemental analysis between Riesling wine samples with and without apple juice adulteration (↓, decrease; ↑, increase).

Element	Median in Unadulterated Wine	Median in Adulterated Wine	Direction	*p*-Value	Holm-Adjusted *p*-Value	Significant After Holm Correction
Al	7.815	6.116	↓	0.0002	0.0017	YES
Ba	0.180	0.164	↓	0.0043	0.0256	YES
Ca	104.5	88.85	↓	0.0001	0.0012	YES
Cr	0.017	0.021	↑	0.1070	0.3210	NO
Cu	0.322	0.265	↓	0.0001	0.0013	YES
Fe	1.962	1.608	↓	0.0009	0.0068	YES
K	782.4	738.7	↓	0.0001	0.0012	YES
Mg	82.49	68.07	↓	0.0001	0.0012	YES
Mn	1.093	0.976	↓	0.0001	0.0012	YES
Na	34.51	37.75	↑	0.0001	0.0013	YES
Ni	0.025	0.019	↓	0.1510	0.3210	NO
P	158.2	131.7	↓	0.0001	0.0012	YES
Pb	0.023	0.021	↓	0.3890	0.3890	NO
S	153.6	132.5	↓	0.0001	0.0012	YES
Sn	0.181	0.138	↓	0.0001	0.0012	YES
Sr	0.419	0.394	↓	0.0001	0.0012	YES
Ti	0.035	0.025	↓	0.0009	0.0068	YES
V	0.019	0.013	↓	0.0103	0.0513	NO after Holm
Zn	1.040	1.007	↓	0.0413	0.1650	NO after Holm

**Table 7:** Statistically significant differences between samples in elemental analysis for Riesling wine samples depending on the adulteration level (* pHolm < 0.05, ** pHolm < 0.01, *** pHolm < 0.001).

Element	Median L0	Median L5	Median L10	Median L20	Friedman *p*-Value	Significant Pairwise Differences After Holm Correction
Al	7.815	5.708	5.746	6.567	2.63 × 10^−4^	L0–L5 **; L0–L10 ***; L0–L20 **
Ba	0.181	0.161	0.159	0.178	3.81 × 10^−4^	L0–L5 **; L0–L10 **; L10–L20 *
Ca	104.5	95.52	92.15	83.16	2.56 × 10^−9^	all pairwise comparisons ***
Cr	0.017	0.023	0.018	0.018	0.0197	none after Holm correction
Cu	0.322	0.279	0.263	0.241	3.10 × 10^−7^	L0–L5 *; L0–L10 **; L0–L20 ***; L5–L10 *; L5–L20 ***; L10–L20 ***
Fe	1.962	1.676	1.661	1.476	3.17 × 10^−6^	L0–L5 *; L0–L10 **; L0–L20 ***; L5–L20 ***; L10–L20 **
K	782.4	734.8	735.8	749.6	3.17 × 10^−6^	L0–L5 ***; L0–L10 **; L0–L20 *; L5–L10 **; L5–L20 *
Mg	82.49	71.6	69.59	63.52	2.56 × 10^−9^	L0–L5 ***; L0–L10 ***; L0–L20 ***; L5–L10 **; L5–L20 ***; L10–L20 ***
Mn	1.093	1.038	1.006	0.912	1.60 × 10^−9^	all pairwise comparisons ***
Na	34.51	30.69	35.19	44.58	4.94 × 10^−7^	L0–L20 ***; L5–L10 **; L5–L20 ***; L10–L20 ***
Ni	0.024	0.019	0.019	0.019	0.2607	not assessed; Friedman ns
P	158.2	139.2	135.0	121.8	1.60 × 10^−9^	all pairwise comparisons ***
Pb	0.023	0.023	0.023	0.023	0.824	not assessed; Friedman ns
S	153.6	142.7	134.8	120.1	1.60 × 10^−9^	all pairwise comparisons ***
Sn	0.181	0.130	0.126	0.148	2.86 × 10^−5^	L0–L5 ***; L0–L10 ***; L0–L20 **
Sr	0.419	0.415	0.399	0.372	4.78 × 10^−9^	L0–L5 **; L0–L10 ***; L0–L20 ***; L5–L10 **; L5–L20 ***; L10–L20 ***
Ti	0.035	0.028	0.028	0.028	0.005	L0–L5 *; L0–L10 **; L0–L20 **
V	0.019	0.013	0.013	0.013	0.090	not assessed; Friedman ns
Zn	1.040	1.068	1.030	0.892	0.002	L5–L10 **

**Table 8:** pH values obtained for Riesling wines with different levels of apple juice adulteration.

Sample Code	5%	10%	20%	Wine Sample Without Adulteration
1Ra	3.10 ± 0.02	3.11 ± 0.02	3.14 ± 0.03	3.08 ± 0.00
2Ra	3.08 ± 0.03	3.14 ± 0.01	3.15 ± 0.03	3.07 ± 0.00
4Ra	3.15 ± 0.02	3.16 ± 0.01	3.18 ± 0.01	3.12 ± 0.01
5Ra	3.12 ± 0.02	3.17 ± 0.01	3.23 ± 0.00	3.09 ± 0.01
20Ra	3.17 ± 0.03	3.19 ± 0.02	3.19 ± 0.01	3.13 ± 0.00
1Rb	3.11 ± 0.00	3.14 ± 0.02	3.18 ± 0.02	3.09 ± 0.02
2Rb	3.13 ± 0.02	3.15 ± 0.01	3.21 ± 0.02	3.10 ± 0.01
4Rb	3.12 ± 0.02	3.16 ± 0.01	3.20 ± 0.03	3.08 ± 0.00
5Rb	3.14 ± 0.02	3.18 ± 0.02	3.22 ± 0.03	3.11 ± 0.00
20Rb	3.09 ± 0.01	3.12 ± 0.02	3.16 ± 0.01	3.07 ± 0.00
1Rc	3.12 ± 0.03	3.15 ± 0.02	3.19 ± 0.02	3.09 ± 0.01
2Rc	3.13 ± 0.01	3.16 ± 0.00	3.2 ± 0.01	3.10 ± 0.02
4Rc	3.10 ± 0.00	3.14 ± 0.00	3.18 ± 0.03	3.08 ± 0.00
5Rc	3.14 ± 0.00	3.17 ± 0.02	3.23 ± 0.00	3.11 ± 0.01
20Rc	3.11 ± 0.01	3.15 ± 0.01	3.19 ± 0.00	3.09 ± 0.01
pH value of apple juice		3.57 ± 0.04		

**Table 9:** Brief characteristics of examined wine samples.

Number of Samples	Grape Variety	Country of Origin
15	Riesling	Germany
15	Chardonnay	Australia (3); Hungary (3); Italy (6)
15	Sauvignon Blanc	Spain (3); Chile (3); Germany (3); Hungary (3); Australia (3)
6	Gewürztraminer	Germany (3); Moldova (3)
3	Solaris	Poland (3)
3	Soave	Italy (3)
3	Muscato	USA (3)

**Table 10:** Basic validation parameters obtained for each analyte for the developed method (*n*, number of standards in three replicates, R^2^, coefficient of determination, CRM’s values).

Element	*n*	R^2^	LOD [mg/L]	LOQ [mg/L]	CRM (TMDA 54.6, * NCS ZC 8100 2b)		THX012RM	
					Certified Value	Obtained Value	Certified Value	Obtained Value
Ag	3	0.999	0.007	0.020	12.90 ± 1.100 μg/L	12.14 ± 0.902 μg/L	-	-
Al		0.999	0.019	0.036	0.389 ± 0.028 mg/L	0.379 ± 0.015 mg/L	-	-
Ba		1.000	0.003	0.01	0.324 ± 0.019 mg/L	0.325 ± 0.014 mg/L	-	-
Ca		1.000	0.033	0.099	* 1537 ± 68.00 μg/mL	1581 ± 48.82 μg/mL	-	-
Cd		0.999	0.0003	0.001	156.0 ± 8.000 μg/L	167.6 ± 6.084 μg/L	72.00 ± 2.200 mg/L	68.75 ± 0.050 mg/L
Co		0.999	0.0003	0.001	305.0 ± 20.00 μg/L	300.4 ± 20.16 μg/L	-	-
Cr		1.000	0.001	0.003	0.421 ± 0.022 mg/L	0.439 ± 0.008 mg/L	-	-
Cu		1.000	0.003	0.009	0.393 ± 0.026 mg/L	0.385 ± 0.019 mg/L	187.0 ± 6.000 mg/L	192.8 ± 0.050 mg/L
Fe		1.000	0.024	0.071	0.367 ± 0.023 mg/L	0.377 ± 0.018 mg/L	1460 ± 70.00 mg/L	1462 ± 42.00 mg/L
K		1.000	0.027	0.082	* (14.40) μg/mL	14.77 ± 0.814 μg/mL	-	-
Mg		0.999	0.005	0.015	* 248.0 ± 14.00 μg/mL	240.1 ± 11.85 μg/mL	-	-
Mn		0.999	0.002	0.004	274.0 ± 13.00 μg/L	271.3 ± 10.24 μg/L	-	-
Mo		0.999	0.007	0.021	292.0 ± 20.00 μg/L	268.8 ± 13.14 μg/L	-	-
Na		1.000	0.051	0.153	* 445.0 ± 40.00 μg/mL	440.7 ± 40.04 μg/mL	-	-
Ni		0.999	0.002	0.006	323.0 ± 19.00 μg/L	297.1 ± 28.39 μg/L	-	-
P		1.000	0.003	0.009	* 174.0 ± 43.00 μg/mL	166.3 ± 9.155 μg/g	-	-
Pb		0.999	0.001	0.003	490.0 ± 30.00 μg/L	457.1 ± 30.03 μg/L	162.0 ± 4.000 mg/L	156.3 ± 1.000 mg/L
S		0.999	0.029	0.087	* (4.620) %	4.404 ± 0.09%	-	-
Sb		0.999	0.005	0.015	* 120.0 ± 20.00 ng/mL	122.1 ± 4.755 ng/mL	-	-
Sn		0.999	0.003	0.009	45.30 ± 3.100 μg/L	46.59 ± 2.180 μg/L	-	-
Sr		1.000	0.005	0.015	0.573 ± 0.031 mg/L	0.513 ± 0.044 mg/L	-	-
Ti		1.000	0.005	0.015	0.032 ± 0.003 mg/L	0.033 ± 0.001 mg/L	-	-
V		1.000	0.002	0.006	(0.089) μg/L	0.104 ± 0.005 μg/L	-	-
Zn		0.998	0.024	0.072	0.540 ± 0.035 mg/L	0.572 ± 0.037 mg/L	-	-

* value for NCS ZC 8100 2b; (…)—reference only.

## Data Availability

The original contributions presented in the study are included in the article/Supplementary Material, further inquiries can be directed to the corresponding author.

## Supplementary Materials

The following supporting information can be downloaded at https://www.mdpi.com/article/10.3390/molecules31203655/s1: Figure S1: Projection of Riesling wine samples (*n* = 60) onto the PCA factor plane based on the full elemental data set, grouped according to apple juice adulteration level; Table S1: Holm-adjusted *p*-values for pairwise comparisons between apple juice adulteration levels obtained using post hoc Wilcoxon signed-rank tests; Table S2: Pearson correlation matrix for all analyzed elements in wine and adulterated samples; Table S3: Eigenvalues and explained variance of principal components derived from the elemental correlation matrix; Table S4: Factor loadings for all principal components obtained from PCA of the reduced elemental data set; Table S5: Detailed information on the authentic white wine samples included in the study.

## Abbreviations

The following abbreviations are used in this manuscript:

ICP-OES	Inductively Coupled Plasma Optical Emission Spectrometry
ICP-MS	Inductively Coupled Plasma Mass Spectrometry
CVAAS	Cold Vapor Atomic Absorption Spectroscopy
PCA	Principal component analysis
OIV	International Organisation of Vine and Wine
LOD	Limit of detection
LOQ	Limit of quantification
