# Supplementary material for: Elemental Changes in Riesling Wines Following Controlled Apple Juice Addition: An ICP–OES-Based Model Study
Source: Molecules. 2026 Oct 13;31(20):3655. doi: 10.3390/molecules31203655 (PMC13707753; doi:10.3390/molecules31203655)
Supplement: Supplementary file 1 [file molecules-31-03655-s001.zip › molecules-4538431-supplementary.pdf]

Table S1. Holm-adjusted p-values for pairwise comparisons between apple juice adulteration levels obtained using post hoc Wilcoxon signed-rank tests

Pairwise comparison	Al	Ba	Ca	Cr	Cu	Fe	K	Mg	Mn	Na
**L0–L5**	**0.0021**	**0.0059**	**0.0004**	0.0747	**0.0205**	**0.0081**	**0.0004**	**0.0004**	**0.0004**	0.1460
**L0–L10**	**0.0004**	**0.0059**	**0.0004**	0.3650	**0.0046**	**0.0085**	**0.0043**	**0.0004**	**0.0004**	0.1688
**L0–L20**	**0.0046**	0.7183	**0.0004**	0.7935	**0.0004**	**0.0006**	**0.0251**	**0.0004**	**0.0004**	**0.0004**
**L5–L10**	0.9086	0.7197	**0.0006**	0.3650	**0.0205**	0.1688	**0.0081**	**0.0015**	**0.0004**	**0.0060**
**L5–L20**	0.9086	0.1060	**0.0004**	0.6233	**0.0004**	**0.0004**	**0.0251**	**0.0004**	**0.0004**	**0.0004**
**L10-L20**	0.7571	**0.0105**	**0.0004**	0.7935	**0.0005**	**0.0081**	0.0946	**0.0004**	**0.0004**	**0.0004**

Pairwise comparison	Ni	P	Pb	S	Sn	Sr	Ti	V	Zn
**L0–L5**	1.0000	**0.0004**	1.0000	**0.0004**	**0.0009**	**0.0012**	**0.0215**	0.3210	0.8904
**L0–L10**	0.9820	**0.0004**	1.0000	**0.0004**	**0.0004**	**0.0004**	**0.0094**	0.2549	0.1279
**L0–L20**	0.2120	**0.0004**	1.0000	**0.0004**	**0.0034**	**0.0004**	**0.007**	**0.0379**	0.1414
**L5–L10**	1.0000	**0.0009**	1.0000	**0.0004**	0.7788	**0.0012**	0.4061	0.5048	**0.0070**
**L5–L20**	0.5350	**0.0004**	1.0000	**0.0004**	0.7788	**0.0004**	0.4586	0.0513	0.2498
**L10-L20**	1.0000	**0.0004**	1.0000	**0.0004**	0.4061	**0.0004**	0.7615	0.5048	0.2498

Table S2. Pearson correlation matrix for all analyzed elements in wine and adulterated samples

Variable	Al	Ba	Ca	Cr	Cu	Fe	K	Mg	Mn	Na	Ni	P	Pb	S	Sn	Sr	Ti	V	Zn
**Al**	1.000	0.558	0.449	0.116	0.134	0.319	0.126	0.373	0.146	0.165	-0.164	0.279	0.100	0.481	0.982	0.184	0.670	0.263	0.175
**Ba**	0.558	1.000	0.261	0.163	0.041	0.238	0.160	-0.107	-0.365	0.380	-0.160	0.643	0.211	-0.132	0.516	-0.353	0.532	0.330	-0.170
**Ca**	0.449	0.261	1.000	-0.117	0.047	0.393	0.382	0.761	0.369	-0.607	0.029	0.727	-0.299	0.805	0.438	-0.034	0.597	0.606	0.249
**Cr**	0.116	0.163	-0.117	1.000	0.081	0.065	-0.220	-0.195	-0.157	0.048	-0.098	-0.006	-0.049	-0.088	0.100	0.040	-0.017	0.079	-0.037
**Cu**	0.134	0.041	0.047	0.081	1.000	0.856	-0.767	-0.394	-0.664	0.121	-0.184	0.129	-0.269	0.257	0.118	0.823	0.184	-0.035	0.204
**Fe**	0.319	0.238	0.393	0.065	0.856	1.000	-0.469	-0.085	-0.491	-0.007	0.019	0.432	-0.259	0.459	0.288	0.645	0.449	0.220	0.261
**K**	0.126	0.160	0.382	-0.220	-0.767	-0.469	1.000	0.674	0.670	-0.163	0.176	0.263	0.115	0.123	0.147	-0.679	0.154	0.355	-0.106
**Mg**	0.373	-0.107	0.761	-0.195	-0.394	-0.085	0.674	1.000	0.861	-0.588	0.141	0.272	-0.090	0.762	0.405	-0.145	0.344	0.459	0.308
**Mn**	0.146	-0.365	0.369	-0.157	-0.664	-0.491	0.670	0.861	1.000	-0.492	0.183	-0.160	0.059	0.468	0.197	-0.256	0.013	0.207	0.228
**Na**	0.165	0.380	-0.607	0.048	0.121	-0.007	-0.163	-0.588	-0.492	1.000	-0.132	-0.282	0.588	-0.609	0.162	0.029	-0.010	-0.337	-0.053
**Ni**	-0.164	-0.160	0.029	-0.098	-0.184	0.019	0.176	0.141	0.183	-0.132	1.000	-0.049	-0.001	0.009	-0.158	-0.125	-0.095	-0.026	0.039
**P**	0.279	0.643	0.727	-0.006	0.129	0.432	0.263	0.272	-0.160	-0.282	-0.049	1.000	-0.202	0.343	0.257	-0.278	0.503	0.612	-0.066
**Pb**	0.100	0.211	-0.299	-0.049	-0.269	-0.259	0.115	-0.090	0.059	0.588	-0.001	-0.202	1.000	-0.336	0.134	-0.242	-0.051	-0.092	0.427
**S**	0.481	-0.132	0.805	-0.088	0.257	0.459	0.123	0.762	0.468	-0.609	0.009	0.343	-0.336	1.000	0.499	0.439	0.450	0.454	0.436
**Sn**	0.982	0.516	0.438	0.100	0.118	0.288	0.147	0.405	0.197	0.162	-0.158	0.257	0.134	0.499	1.000	0.187	0.614	0.294	0.211
**Sr**	0.184	-0.353	-0.034	0.040	0.823	0.645	-0.679	-0.145	-0.256	0.029	-0.125	-0.278	-0.242	0.439	0.187	1.000	0.068	-0.153	0.363
**Ti**	0.670	0.532	0.597	-0.017	0.184	0.449	0.154	0.344	0.013	-0.010	-0.095	0.503	-0.051	0.450	0.614	0.068	1.000	0.421	0.092
**V**	0.263	0.330	0.606	0.079	-0.035	0.220	0.355	0.459	0.207	-0.337	-0.026	0.612	-0.092	0.454	0.294	-0.153	0.421	1.000	0.082
**Zn**	0.175	-0.170	0.249	-0.037	0.204	0.261	-0.106	0.308	0.228	-0.053	0.039	-0.066	0.427	0.436	0.211	0.363	0.092	0.082	1.000

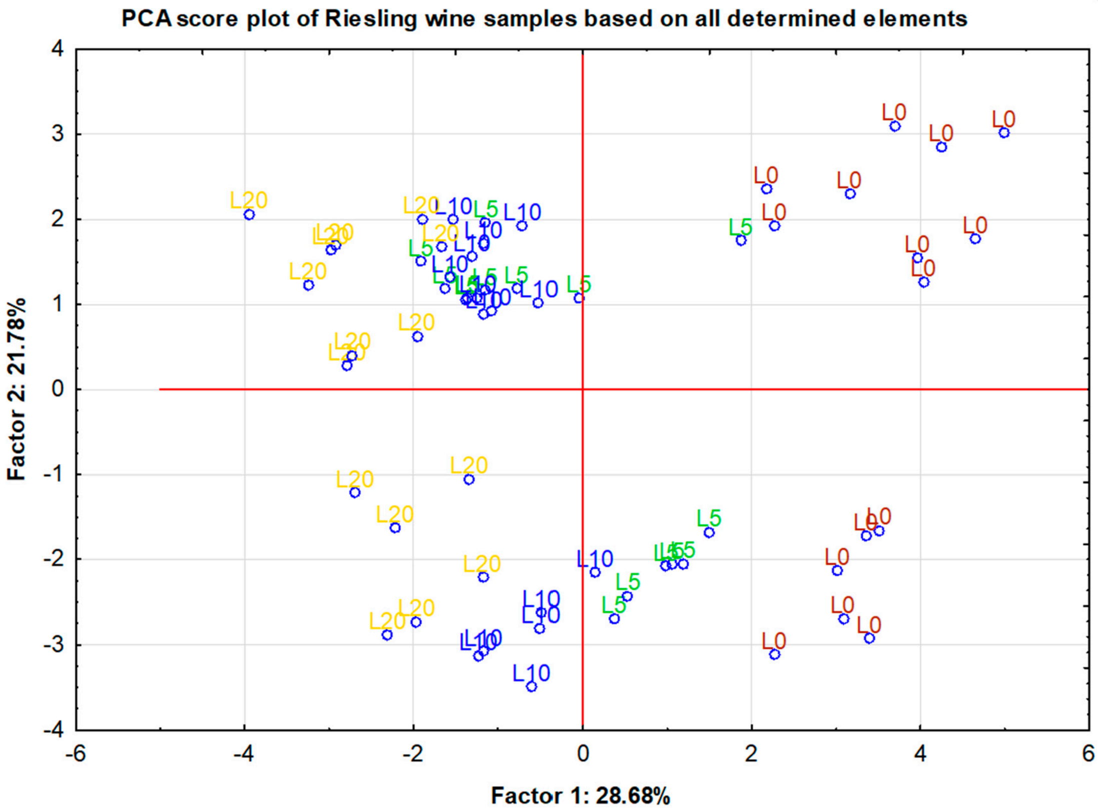

Figure S1. Projection of Riesling wine samples (n = 60) onto the PCA factor plane based on the full elemental data set, grouped according to apple juice adulteration level

Table S3. Eigenvalues and explained variance of principal components derived from the elemental correlation matrix

Value number	Eigenvalues of correlation matrix, and related statistics			
	Eigenvalue	% Total	Cumulative	Cumulative
1	3.778	41.98	3.780	41.98
2	3.164	35.16	6.940	77.14
3	1.387	15.41	8.330	92.55
4	0.506	5.619	8.835	98.17
5	0.086	0.954	8.921	99.12
6	0.049	0.543	8.970	99.67
7	0.017	0.187	8.987	99.85
8	0.010	0.110	8.997	99.96
9	0.003	0.035	9.000	100.0

Table S4. Factor loadings for all principal components obtained from PCA of the reduced elemental data set

Variable	Factor coordinates of the variables, based on correlations								
	Factor 1	Factor 2	Factor 3	Factor 4	Factor 5	Factor 6	Factor 7	Factor 8	Factor 9
**Ca**	0.902	-0.273	0.272	0.086	0.016	-0.172	-0.026	0.012	-0.010
**Cu**	-0.207	-0.962	-0.103	-0.067	-0.071	-0.047	0.097	0.009	0.006
**Fe**	0.110	-0.953	0.111	0.108	0.227	0.058	-0.004	0.008	0.001
**Mg**	0.936	0.210	-0.150	0.225	0.018	0.015	0.038	-0.073	0.004
**Mn**	0.698	0.574	-0.398	0.128	0.031	0.038	0.044	0.055	-0.023
**P**	0.485	-0.322	0.799	0.025	-0.110	0.096	0.004	0.002	-0.019
**S**	0.849	-0.425	-0.277	0.086	-0.092	0.048	-0.036	0.029	0.035
**Sr**	-0.082	-0.773	-0.622	0.011	-0.072	0.014	-0.045	-0.021	-0.031
**Na**	-0.764	-0.011	0.084	0.638	-0.043	-0.015	-0.003	0.009	0.001

Table S5. Detailed information on the authentic white wine samples included in the study

No.	Sample code	Grape variety	Country of origin	Region of origin	Wine style	Vintage	Alcohol content [% v/v]
1	1Ra	Riesling	Germany	Mosel	semi-sweet	2023	10.5
2	1Rb	Riesling	Germany	Mosel	semi-sweet	2023	10.5
3	1Rc	Riesling	Germany	Mosel	semi-sweet	2023	10.5
4	2Ra	Riesling	Germany	Mosel	semi-sweet	2023	10.5
5	2Rb	Riesling	Germany	Mosel	semi-sweet	2023	10.5
6	2Rc	Riesling	Germany	Mosel	semi-sweet	2023	10.5
7	3Ga	Gewürztraminer	Germany	Palatinate	semi-sweet	2023	11
8	3Gb	Gewürztraminer	Germany	Palatinate	semi-sweet	2023	11
9	3Gc	Gewürztraminer	Germany	Palatinate	semi-sweet	2023	11
10	4Ra	Riesling	Germany	Palatinate	semi-dry	2023	10
11	4Rb	Riesling	Germany	Palatinate	semi-dry	2023	10
12	4Rc	Riesling	Germany	Palatinate	semi-dry	2023	10
13	5Ra	Riesling	Germany	Palatinate	semi-dry	2023	10
14	5Rb	Riesling	Germany	Palatinate	semi-dry	2023	10
15	5Rc	Riesling	Germany	Palatinate	semi-dry	2023	10
16	6Sa	Solaris	Poland	West Pomerania	dry	2023	12.5
17	6Sb	Solaris	Poland	West Pomerania	dry	2023	12.5
18	6Sc	Solaris	Poland	West Pomerania	dry	2023	12.5

19	7SBa	Sauvignon Blanc	Hungary	Mátra	dry	2021	12
20	7SBb	Sauvignon Blanc	Hungary	Mátra	dry	2021	12
21	7SBc	Sauvignon Blanc	Hungary	Mátra	dry	2021	12
22	8Ca	Chardonnay	Hungary	Szekszárd	dry	2023	14
23	8Cb	Chardonnay	Hungary	Szekszárd	dry	2023	14
24	8Cc	Chardonnay	Hungary	Szekszárd	dry	2023	14
25	9Ca	Chardonnay	Australia	South Eastern Australia	dry	2022	12.5
26	9Cb	Chardonnay	Australia	South Eastern Australia	dry	2022	12.5
27	9Cc	Chardonnay	Australia	South Eastern Australia	dry	2022	12.5
28	10SBa	Sauvignon Blanc	Australia	South Eastern Australia	semi-dry	2023	11.5
29	10SBb	Sauvignon Blanc	Australia	South Eastern Australia	semi-dry	2023	11.5
30	10SBc	Sauvignon Blanc	Australia	South Eastern Australia	semi-dry	2023	11.5
31	11Ga	Gewürztraminer	Moldova	not declared	semi-dry	2024	14
32	11Gb	Gewürztraminer	Moldova	not declared	semi-dry	2024	14
33	11Gc	Gewürztraminer	Moldova	not declared	semi-dry	2024	14
34	12Ca	Chardonnay	Italy	Garda	not declared	2023	12
35	12Cb	Chardonnay	Italy	Garda	not declared	2023	12
36	12Cc	Chardonnay	Italy	Garda	not declared	2023	12
37	13Ca	Chardonnay	Italy	Emilia-Romagna	dry	not declared	12
38	13Cb	Chardonnay	Italy	Emilia-Romagna	dry	not declared	12
39	13Cc	Chardonnay	Italy	Emilia-Romagna	dry	not declared	12
40	14SVa	Soave	Italy	Veneto	dry	2023	not declared
41	14SVb	Soave	Italy	Veneto	dry	2023	not declared
42	14SVc	Soave	Italy	Veneto	dry	2023	not declared
43	15SBa	Sauvignon Blanc	Germany	Palatinate	sweet	not declared	0
44	15SBb	Sauvignon Blanc	Germany	Palatinate	sweet	not declared	0
45	15SBc	Sauvignon Blanc	Germany	Palatinate	sweet	not declared	0
46	16SBa	Sauvignon Blanc	Chile	Central Valley	not declared	2024	not declared
47	16SBb	Sauvignon Blanc	Chile	Central Valley	not declared	2024	not declared
48	16SBc	Sauvignon Blanc	Chile	Central Valley	not declared	2024	not declared
49	17Ma	Moscato	United States	New York	sweet	not declared	10
50	17Mb	Moscato	United States	New York	sweet	not declared	10

**51**	**17Mc**	**Moscato**	**United States**	**New York**	**sweet**	**not declared**	**10**
**52**	**18Ca**	**Chardonnay**	**Italy**	**Trentino**	**dry**	**2024**	**12**
**53**	**18Cb**	**Chardonnay**	**Italy**	**Trentino**	**dry**	**2024**	**12**
**54**	**18Cc**	**Chardonnay**	**Italy**	**Trentino**	**dry**	**2024**	**12**
**55**	**19SBa**	**Sauvignon Blanc**	**Spain**	**not declared**	**not declared**	**not declared**	**0**
**56**	**19SBb**	**Sauvignon Blanc**	**Spain**	**not declared**	**not declared**	**not declared**	**0**
**57**	**19SBc**	**Sauvignon Blanc**	**Spain**	**not declared**	**not declared**	**not declared**	**0**
**58**	**20Ra**	**Riesling**	**Germany**	**Palatinate**	**semi-dry**	**2023**	**11.5**
**59**	**20Rb**	**Riesling**	**Germany**	**Palatinate**	**semi-dry**	**2023**	**11.5**
**60**	**20Rc**	**Riesling**	**Germany**	**Palatinate**	**semi-dry**	**2023**	**11.5**
